# Editorial: Mesenchymal stem cells and derived extracellular vesicles as next-generation biological drugs for tissue regeneration

**DOI:** 10.3389/fphar.2025.1770314

**Published:** 2026-01-08

**Authors:** Silvia Barbon, Antara Banerjee, Andrea Porzionato

**Affiliations:** 1 Department of Neuroscience, Section of Human Anatomy, University of Padua, Padua, Italy; 2 Foundation for Biology and Regenerative Medicine, Tissue Engineering and Signaling – TES Onlus, Padova, Italy; 3 Faculty of Allied Health Sciences, Chettinad Academy of Research and Education (CARE), Chettinad Hospital and Research Institute (CHRI), Chennai, India

**Keywords:** biological drug, cell therapy, extracellular vesicles, mesenchymal stem cells, regenerative medicine, tissue regeneration

**FIGURE 1:**
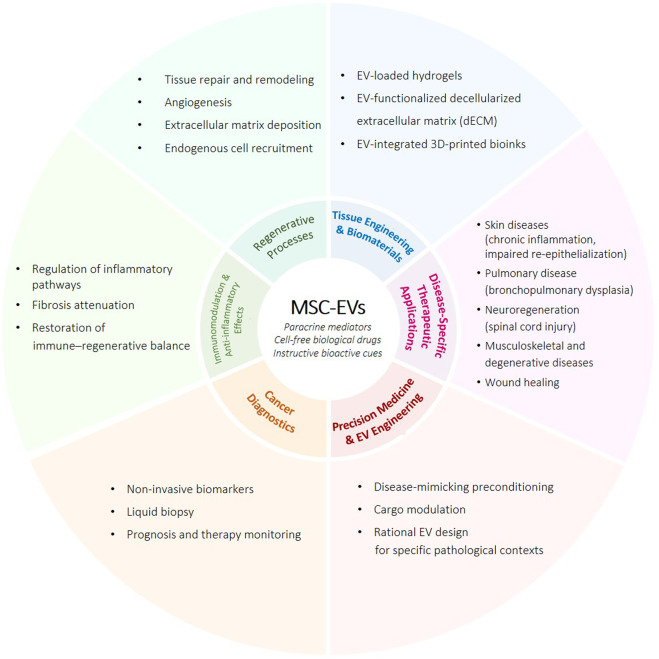
Overview of the biological functions and translational applications of mesenchymal stem cell–derived extracellular vesicles (MSC-EVs). (Created with BioRender.com).

Mesenchymal stem cells (MSCs) have emerged as a potent tool in regenerative medicine owing to their capacity for self-renewal, multipotency, and immunomodulation (Barbon et al., 2022; Barbon et al., 2021; Di Liddo et al., 2016; Di Liddo et al., 2014). The therapeutic efficacy of MSCs derived from different tissues (i.e., bone marrow, adipose tissue, peripheral blood, umbilical cord, placenta and amniotic membrane/fluid) has been rigorously investigated in preclinical models and clinical trials across an extensive range of human diseases, comprising autoimmune disorders, neurodegenerative diseases, inflammatory disorders, and musculoskeletal injuries (Han et al., 2025). Initially ascribed to direct cellular differentiation or cell-to-cell communication with immune cells, the therapeutic role of MSCs is now recognized to be largely mediated through paracrine modulation of the tissue microenvironment (Patel et al., 2025). The key effectors of this activity are extracellular vesicles (EVs) derived from MSCs (e.g., exosomes and microvesicles), which serve as natural nanocarriers of bioactive molecules with trophic, immunomodulatory, and pro-regenerative effects. This could indicate a conceptual shift from cell-based transplantation to cell-derived, cell-free treatments, leading to new therapeutic approaches focused on the precise regulation of inflammatory pathways, the enhancement of tissue repair, and the restoration of immune-regenerative equilibrium (Figure 1).

In this context, recent evidence also acknowledges the therapeutic use of MSC-EVs as integrative platforms for personalized medicine, especially for cancer diagnosis and treatment. Notably, EVs have recently been proposed as key mediators of tumor-microenvironment interplay, unveiling their value as non-invasive biomarkers in oncology. The review by Bernad et al. (2025) highlights how tumor-derived EVs carry disease-specific molecular signatures (i.e., proteins, lipids, and nucleic acids such as miRNAs) that can be leveraged for liquid biopsy, cancer diagnosis, prognosis, and monitoring of therapeutic response.

In this Research Topic, some works examine how the therapeutic potential of MSCs can be refined and controlled through EV-based approaches. Gong et al. present a comprehensive analysis of adipose-derived MSC (ADMSC) exosomes, exploring their role in modulating inflammatory pathways and supporting tissue remodeling in degenerative and immune-mediated conditions (Gong et al.). Besides underlining exosome potential as programmable drug carriers which can be engineered for enhanced targeting and synergy, the authors point out that their clinical translation depends on overcoming technical and regulatory barriers. In line with this work, Gurney et al. demonstrate how the selective enhancement of EV cargo can promote wound healing, showing that vesicles are not only natural mediators of tissue regeneration but can be specifically engineered to activate key biological pathways (Gurney et al.). Both the studies highlight a major trend in this research field: EVs are undergoing a fundamental redefinition, from passive biomarkers of physiological states to actively engineered, customizable therapeutic platforms.

The integration of MSCs with biomaterials is another crucial aspect of this translational shift. Barbon et al. discuss hydrogels as delivery platforms that prolong MSC viability, locally concentrate their paracrine secretion, and improve functional outcomes in several tissues, bridging preclinical applications and early clinical use (Barbon et al.). Notably, the authors present hydrogels not as inert carriers, but as bioactive and cell-instructive scaffolds capable of influencing cellular behavior via mechanotransduction, a critical insight for engineering mechanically dynamic tissues. Interestingly, a growing body of evidence shows that hydrogels and scaffold-based systems are also effective in the targeted delivery of MSC-EVs, enhancing localized retention, sustaining cargo release and avoiding rapid clearance (Chen et al., 2021; Hu et al., 2020; Zhang et al., 2018; Liu et al., 2017). Starting form this concept, MSC-EVs are overcoming the boundaries of cell-based approaches, being increasingly investigated as bioactive tools that reshape structural biomaterials into instructive cell-free constructs. Indeed, MSC-EVs are now directly integrated into hydrogels, decellularized extracellular matrices, and 3D-printed bioinks to provide sustained and spatially controlled delivery of vesicle cargo, regulate endogenous cell recruitment, guide matrix deposition, and promote neo-vascularization for functional tissue formation. Based on this approach, EVs are used not only as therapeutic agents but as bioactive cues that functionalize biomaterials, supporting the design of next-generation engineered scaffolds (Lu et al., 2022).

The therapeutic potential of MSC and EV treatments is increasingly being validated within the complex pathophysiology of organ-specific diseases. Matwiejuk et al. address dermatological applications considering skin diseases like atopic dermatitis, psoriasis, systemic sclerosis, contact dermatitis, graft-versus-host disease, alopecia areata, and systemic lupus erythematosus. In these conditions, chronic inflammation, fibrosis, and impaired re-epithelialization are crucial pathological features that require effective therapeutic intervention (Matwiejuk et al.). The authors’ analysis supports a role for MSC-EVs as multi-target regulators capable of integrating immune modulation with structural repair. Ye et al., further expand the paradigm by demonstrating that preconditioning MSC-EVs under disease-mimicking environments enhances therapeutic precision, reinforcing the concept that regenerative interventions should reflect the complexity of pathological niches. Consequently, the field is evolving from indiscriminate administration toward the rational design of EVs preconditioned for specific disorders, such as systemic inflammation.

Large-scale analytical studies try to define emerging clinical priorities. The bibliometric analysis by Bai and Xin trace two decades of research on MSCs in neonatal bronchopulmonary dysplasia (BPD), documenting a clear thematic shift from whole-cell therapies towards MSC-derived exosomes as a dominant cell-free approach after 2018 (Bai and Xin). Although preclinical efficacy of MSC-EVs in BPD has been extensively reported in the scientific literature (Bisaccia et al., 2024; Porzionato et al., 2021; Porzionato et al., 2019), this work outlines a significant translational gap, as only 8.2% of studies report clinical outcomes. This suggests the need for future research advancement to bridge preclinical findings with standardized clinical trials and mechanistic studies. Yang et al. present a complementary bibliometric overview in degenerative joint and bone diseases, revealing increasing attention towards the therapeutic use of MSC-EVs to repair cartilage and bone, to regulate the immune system, and to act as targeted delivery vehicles for advanced regenerative strategies (Yang et al.).

Neuroregeneration is represented by Cipriano et al., who provide preclinical evidence that human umbilical cord MSC-EVs improve survival, motor function, and neuroinflammatory parameters in a rat model of spinal cord injury (Cipriano et al.). Beyond functional benefits, biodistribution analyses confirm selective EV retention at the injury site, reinforcing their suitability as targeted modulators of neuroinflammation and glial scarring. This work demonstrates how EVs can overcome limitations of direct MSC transplantation in the central nervous system, such as low engraftment, embolic risk, and safety concerns associated with proliferating cells.

Finally, the contribution by Shibili P. et al., broadens the scope beyond organ repair by examining the molecular interplay between endoplasmic reticulum (ER) stress and exosome-mediated communication in inflammatory bowel disease. Their review highlights a bidirectional regulatory axis in which ER stress alters vesicle biogenesis and cargo, while stress-modified vesicles propagate inflammatory signaling across epithelial and immune cells. This mechanistic insight positions EVs not only as therapeutic tools but also as pathogenic mediators, reinforcing the need for nuanced targeting strategies that distinguish beneficial from deleterious signaling (Shibili P. et al.).

Collectively, the articles in this Research Topic explore several themes that define the current trends of MSC-based regenerative medicine. First, therapeutic action is increasingly attributed to highly orchestrated paracrine networks rather than direct cell replacement. Second, EVs provide a scalable, cell-free platform with minimized safety risks and greater engineering potential compared to live cell therapies. Third, disease specificity achieved through preconditioning, cargo modulation, or biomaterial delivery appears essential to achieving clinically significant outcomes. Finally, bibliometric analyses are laying the groundwork for regulatory approval by clarifying therapeutic targets, methodological gaps, and translational priorities.

Future efforts should focus on standardizing EV isolation protocols, defining potency assays, improving in-tissue retention, and developing regulatory standards for the production and use of MSCs and derived EVs as effective biological drugs. As the field progresses, the integration of omics-based characterization, mechanobiology, and smart biomaterials will enable a new generation of precision therapeutic tools where MSCs represent natural bio-factory systems and derived EVs act as tunable agents that can modulate and heal the pathological microenvironments.
